# A decision-making model for prediction of a stable disease course in chronic hepatitis B patients

**DOI:** 10.1038/s41598-023-50460-2

**Published:** 2023-12-27

**Authors:** Imri Ofri, Noam Peleg, Moshe Leshno, Amir Shlomai

**Affiliations:** 1 Department of Medicine D and the Laboratory of Liver Research, Rabin Medical Center, Beilinson Hospital, 39 Jabotinsky Street, Petah-Tikva, Israel; 2 The Faculty of Medicine, Tel-Aviv University, Tel-Aviv, Israel; 3 The Institute of Gastroenterology, Beilinson Hospital, Petah-Tikva, Israel; 4 The Coller Faculty of Management, Tel-Aviv University, Tel-Aviv, Israel; 5 The Liver Institute, Beilinson Hospital, Petah-Tikva, Israel

**Keywords:** Hepatology, Hepatitis B

## Abstract

Patients with chronic hepatitis B (CHB) are regularly monitored for HBV DNA and liver enzymes in order to assess disease progression and the need for antiviral therapy. Identifying patients with a stable course of disease can potentially prolong the intervals between visits, withhold unnecessary tests and save money. Accordingly, we aimed to find predictors for a stable disease course in patients with CHB. 579 patients with CHB, who were followed in a tertiary referral center between January 2004–December 2018, were retrospectively analyzed. Patients with low and steady viral load titer (< 2000 IU/ml) and normal ALT levels (< 40 IU/ml) in 6 consecutive clinic encounters were considered to have a stable course of CHB. A stepwise multivariate logistic regression analysis and a decision tree model were used to identify predictors of a stable disease course. Following exclusion of ineligible patients, a total of 220 patients were included in the final analysis. 64/220 patients had a stable disease course. Patients with a stable disease were older (62.99 ± 12.36 Vs. 54.07 ± 13.64, *p* < 0.001) with a higher percentage of women (53% vs. 38%) and had lower baseline levels of AST, ALT and viral load (VL). In a multivariate analysis, age (OR 0.94, 95% CI 0.91–0.98), baseline ALT (OR 1.06, 95% CI 1.01–1.1) and VL (OR 1.05 95% CI 1.02–1.08), were significantly associated with a stable disease. In a decision tree model, patients 46–67 years old, with baseline VL < 149 IU/mL and ALT < 40 IU/mL had the best probability (91%) for a stable disease course over 4.4 ± 2.2 years. We conclude that integrating patients’ age with baseline VL and ALT can predict a stable disease course in patients with CHB off treatment.

## Introduction

Chronic hepatitis B Virus (CHB) infection affects around 270 million people worldwide and may result in cirrhosis, liver failure and hepatocellular carcinoma (HCC)^1,2^. Nucleotide analogs (NAs), the most used anti-viral agents, efficiently inhibit viral replication but do not eliminate the virus due to the persistence of its episomal DNA (cccDNA) in the infected cells’ nuclei^3,4^. Therefore, most HBeAg negative patients treated with NAs require life-long treatment and only the minority achieve a functional cure, defined as HBsAg loss from serum.

Initiation of NAs therapy is currently recommended only for patients with high viral replication and evidence of active inflammation and/or fibrosis^5,6^. Since this group of patients is at a high risk for advanced liver fibrosis and HCC, the incidence of which can be drastically decreased by efficient viral suppression^7,8^. In addition, patients with cirrhosis should be treated with NAs in order to minimize their risk of liver decompensation and death^9^.

Overall, only the minority of CHB patients are eligible for NAs treatment according to the current recommendations. For example, in a large survey of CHB patients in the US, among 9,129 patients who had an adequate evaluation for their disease status, 11.2% and 13.9% were treatment eligible by AASLD or EASL criteria, respectively^10^. Patients defined as having chronic infection, rather than chronic hepatitis, are usually monitored biannually for HBV viral load and for liver enzymes^5,6^, in order to assess disease progression and qualification for anti-viral treatment initiation.

While most previous studies and current guidelines have focused on simple predictors for a severe disease course, which relay mainly on the magnitude of viral replication and liver enzymes, it is not clear yet whether additional factors, such as age, gender or viral genotype should be also incorporated into the decision considerations for treatment initiation^11^, in order to better allocate high-risk patients.

Identifying predictors for a long-lasting stable disease course, that most often do not require initiation of NA treatment, can potentially prolong the intervals between visits, withhold unnecessary tests and save money by economizing management.

The aims of this study were to characterize patients who have a low probability for disease progression and thus for anti-viral treatment, and to identify predictors for a stable disease course in patients with CHB who are currently not treated with anti-viral agents.

## Methods

### Study design

This retrospective study was approved by the local institutional review board (IRB) of Rabin Medical Center (#RMC-19-0643), according to the local regulations and according to the Helsinki declaration. A waiver for patients’ informed consent was obtained by the Rabin Medical Center IRB due to the retrospective nature of the study. Medical records of all patients > 18 years old, referred to the Liver Institute of Rabin Medical Center with a diagnosis of CHB between the years 2004–2018, were screened for inclusion eligibility. Patients’ demographic, clinical and laboratory data were obtained from their electronic records which included hospitalization summaries, blood test results, imaging reports, liver outpatient clinics reports and outpatient visits at the primary care centers. All the patients were followed according to international guidelines. Follow up included periodic measurements of HBV viral load (VL), as well as serum AST, ALT, alkaline phosphatase and GGT, albumin, bilirubin and alpha fetoprotein levels. Liver fibrosis was assessed using baseline FIB-4 score for all patients. A score > 2.65 was considered as a strong predictor for advanced fibrosis.

In addition, in cases that a liver biopsy was obtained during the follow up, documentation of histological examination regarding liver fibrosis was also gathered and was performed by an experienced pathologist. The degree of fibrosis was reported using the Metavir score. A Metavir score above 2 was defined as advanced fibrosis.

As part of the routine follow up, patients have undergone ultrasonographic examination. Patients who had two consecutive sonographic examinations showing hepatic fatty infiltration, were considered as having hepatic steatosis.

Patients with any secondary liver disease at baseline due to other etiology, such as viral hepatitis, liver malignancy, autoimmune hepatitis, alcohol induced hepatitis and fatty liver disease, were excluded. However, any new medical documentation during follow up tests, such as steatosis per ultrasonographic test, did not exclude the patients from analysis.

Patients with prior antiviral treatment, pregnant patients and patients who had liver transplantation were also excluded. Patients who were HBeAg positive were excluded.

### Definition of a stable and unstable disease course

Patients were retrospectively divided into two groups: “stable patients” were defined as those with HBV DNA VL < 2000 IU/ml and ALT < 40 IU/L in six consecutive lab results throughout the follow up.

All other patients, who fulfilled the need of 6 consecutive lab results, but did not meet the definition of “stable disease”, were considered as patients with ”unstable disease”. The first, out of 6 consecutive lab results, was considered as the baseline value.

Results of liver tissue biopsies, FIB4 scores or patient’s imaging results were not a part of the stability definitions.

### Statistical analysis

Statistical analysis was performed using SPSS and MATLAB software. The endpoint of this study was to characterize the group of patients with stable course of CHB and to find predictors for mild disease course according to our definition of stable patients as described above. Categorical variables were compared by $\chi$^2^ test, whereas continuous variables were compared with the Student’s t test. Correlation was evaluated by the Pearson correlation coefficient. A 2-sided P value of less than 0.05 was considered statistically significant. A univariate logistic regression analysis was conducted in order to determine variable significance between the two groups. The following variables were included in the analysis: demographics such as age and gender, HBV-DNA, ALT, AST, Alkaline Phosphatase, GGT, albumin, INR, total bilirubin levels, WBC, HbA1c and platelets. A stepwise multivariate analysis was conducted in order to examine whether a combination of variables may have an independent effect on the research endpoint. In this method, the following variables were included: Age, Gender, ALT, HBV-DNA, Albumin, Alkaline Phosphatase, AST, GGT, Platelets and Total bilirubin. Simultaneously, we generated a decision tree model which depicted the probability of being a stable or an unstable patient according to the patient’s demographic information and laboratory tests. The same variables that were chosen in the multivariate analysis were applied in the decision tree. The decision tree model aimed to find the optimal variable combination of laboratory tests for prediction of viral DNA stability. The diagnostic accuracy and ability of both the logistic regression and the decision tree with minimum leaf size of 20 as a constraint to distinguish between stable and unstable patients was investigated by determining the area under the receiver operating characteristic (ROC) curve. In addition, for the decision tree we conducted a tenfold cross validation.

## Results

### Study population and baseline characteristics

**Figure 1:**
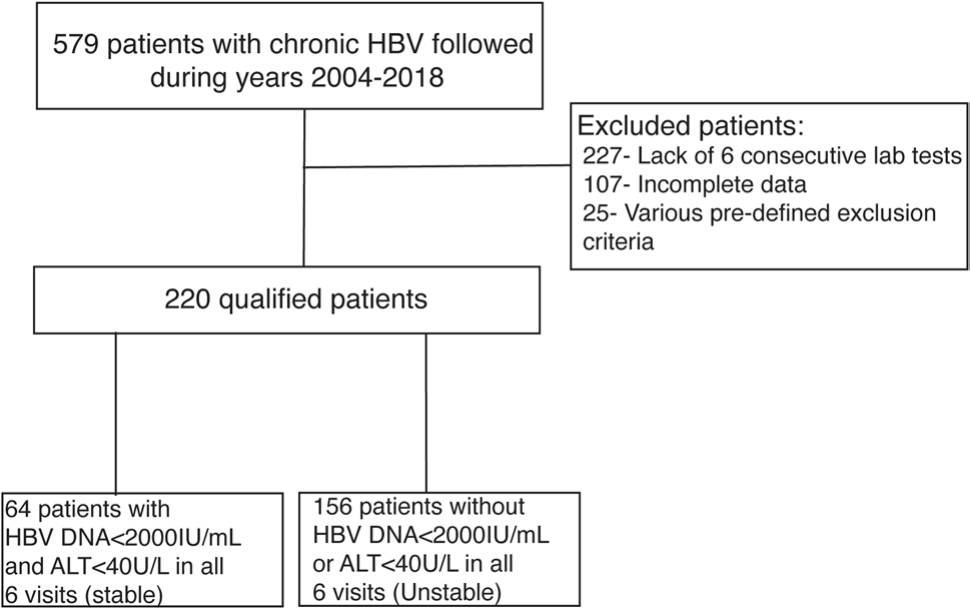
Study flowchart. A study flowchart, showing the various exclusion criteria of 359/579 patients and the categorization of the 220 included patients into “stable” (64) and “unstable” (156).

Medical records of the 579 patients with CHB who were followed at our Liver Institute between the years 2004–2018, were reviewed. 359 participants were excluded, mainly due to insufficient data and due to pre-defined research exclusion criteria. The remaining 220 participants were included in the final analysis (Fig. 1).

Based on our pre-specified definition, 64/220 patients were defined as having a stable disease (“stable”) and the other 156 patients were defined as having an unstable disease (“unstable”).

Follow up time ranged from 2.1 to 12.8 years with an average of 4.4 ± 2.2 years for the whole study population. The average follow up time of the stable group was 4.3 ± 2.15 years and for the unstable group was 4.5 ± 2.3 years (p = 0.57).

Table 1 outlines the baseline characteristics of the study population according to their status of disease stability.

**Table 1:** Baseline characteristics of the cohort, including patient’s demographic and clinical characteristics.

	Stable (n = 64)	Unstable (n = 156)	P-value
Age (years)	62.99 (± 12.36)	54.07 (± 13.64)	< 0.001
Gender-male N (%)	30 (46.8%)	97 (62.1%)	< 0.001
Follow up (years)	4.31 (± 2.15)	4.5 (± 2.3)	0.571
BMI	26.41 (± 3.7)	26.99 (± 5)	0.418
Type 2 DM N (%)	11 (17.1%)	31 (19.8%)	0.162
Hypertension N (%)	21 (32.8%)	23 (14.7%)	< 0.001
Hepatitis D positive N (%)	1 (1.5%)	7 (4.4%)	0.093
HBV DNA (IU/ml)	61.5 [0, 392]	2552 [344, 23000]	< 0.001
AST (U/L)	24.43 (± 13.28)	34.41 (± 23.68)	0.001
ALT (U/L)	20.56 (± 7.63)	43.3 (± 45.16)	< 0.001
ALKP (U/L)	81.6 (± 38.37)	74.88 (± 28.29)	0.164
GGT (U/L)	28.88 (± 28.97)	37.75 (± 41.47)	0.16
Albumin (gr/dL)	4.32 (± 0.31)	4.36 (± 0.45)	0.544
Tot-Bilirubin (mg/dL)	0.61 (± 0.24)	0.7 (± 0.32)	0.064
Platelets (K/micl)	216 (± 62.35)	217 (± 62.53)	0.93
INR	1.02 (± 0.38)	1.03 (± 0.11)	0.846
Fib-4	1.4 (± 0.86)	1.5 (± 1.19)	0.52
FIB-4 > 2.65 N (%)	5 (7.8%)	19 (12.1%)	0.003
Alpha-feto protein (ng/ml)	3.49 (± 2.43)	3.52 (± 3.25)	0.954

Normally distributed variables are reported as Mean ± SD or percentage (%). Non -normally distributed variables are reported as Median [inter-quartile range].

Patients with a stable disease were significantly older compared to those with unstable disease (62.99 ± 12.36 vs. 54.07 ± 13.64, respectively, *p* < 0.001), with a lower percentage of male patients (46.8% vs. 62.1%, *p* < 0.001). As expected, both baseline VL (61.1 [0, 392] vs. 2552 [344, 23000]) and ALT levels (20.56 ± 7.63 vs. 43.3 ± 45.16, *p* < 0.001) were lower in the stable group compared to the unstable group.

Of note, although the mean FIB-4 score was not significantly different between the two groups, the percentage of patients with a calculated FIB-4 score > 2.65 was lower in the stable group, compared to the unstable group of patients (7.8% vs. 12.1%, *p* = 0.003). A total of 8 patients had a baseline of FIB4 < 2.65 that progressed to a FIB4 > 2.65 at the end of the study period, 7 of them were categorized as unstable and only 1 belonged to the stable group.

Other baseline clinical and laboratory parameters, such as BMI, the presence of type 2 DM, as well as serum albumin, INR, bilirubin and platelet count were not statistically different between the two groups.

Regarding liver steatosis, even though none of the patients had any documented secondary liver disease at baseline (including fatty liver disease), during follow up, 46.8% of the stable group and 42.9% of the unstable group had evidence of liver steatosis per sonography. The percentage of patients with steatosis did not differ significantly between the groups.

### Baseline parameters associated with an unstable CHB course

In a univariate logistic regression analysis (Table 2), older age was a protective factor from an unstable disease course (OR 0.94, 95% CI 0.92–0.97) whereas male gender was associated with an unstable disease (OR 1.86, 95% CI 1.03–3.35). As expected, a higher baseline VL (OR 1.04, 95% CI 1.03–1.06), as well as higher baseline AST (OR 1.05, 95% CI 1.01–1.08) and ALT levels (OR 1.08, 95% CI 1.04–1.12) were associated with an unstable CHB course during follow-up.

**Table 2:** A univariate logistic regression analysis of various baseline parameters and their association with an unstable disease course.

	OR	p-value	95% CI	
Age	0.94	< 0.001	0.92	0.97
Male Gender	1.86	0.03	1.03	3.35
VL (1st visit)	1.04	< 0.001	1.03	1.06
ALT	1.08	< 0.001	1.04	1.12
HbA1C	1.07	0.77	0.66	1.72
ALB	1.28	0.54	0.57	2.84
ALKP	0.99	0.17	0.98	1.00
AST	1.05	< 0.001	1.01	1.08
GGT	1.00	0.17	0.99	1.01
INR	1.19	0.84	0.19	7.28
PLT	0.99	0.93	0.99	1.00
Tot_Bilirubin	2.94	0.06	0.92	9.34
WBC	1.06	0.34	0.93	1.21

We next performed an expanded stepwise multivariate logistic regression analysis adjusting for the following variables: Age, gender, ALT, VL, albumin, ALKP, AST, GGT, platelet count and total bilirubin (Table 3). Older age was found to be a protective factor from an unstable disease course (OR 0.94, 95% CI 0.91–0.98), whereas higher baseline ALT (OR 1.06, 95% CI 1.01–1.1) and VL (OR 1.05, 95% CI 1.02–1.08) were independently associated with an unstable disease course.

**Table 3:** A stepwise multivariate logistic regression analysis showing the major laboratory predictors for characterizing unstable CHB patients. (Sig, Significance; OR, odds ratio; C.I, confidence interval).

	Sig	OR	95% CI for OR	
			Lower	Upper
Age	0.003	0.94	0.91	0.98
ALT	0.005	1.06	1.01	1.10
VL	0.000	1.05	1.02	1.08
Constant	0.046	11.68		

### A decision tree model for prediction of an unstable CHB course

Next, a decision tree model was performed by incorporating the same baseline parameters executed in the stepwise multivariate logistic regression of the study’s population, as follows: Age, gender, ALT, AST, ALKP, GGT, VL, albumin, platelets count and total bilirubin level.

**Figure 2:**
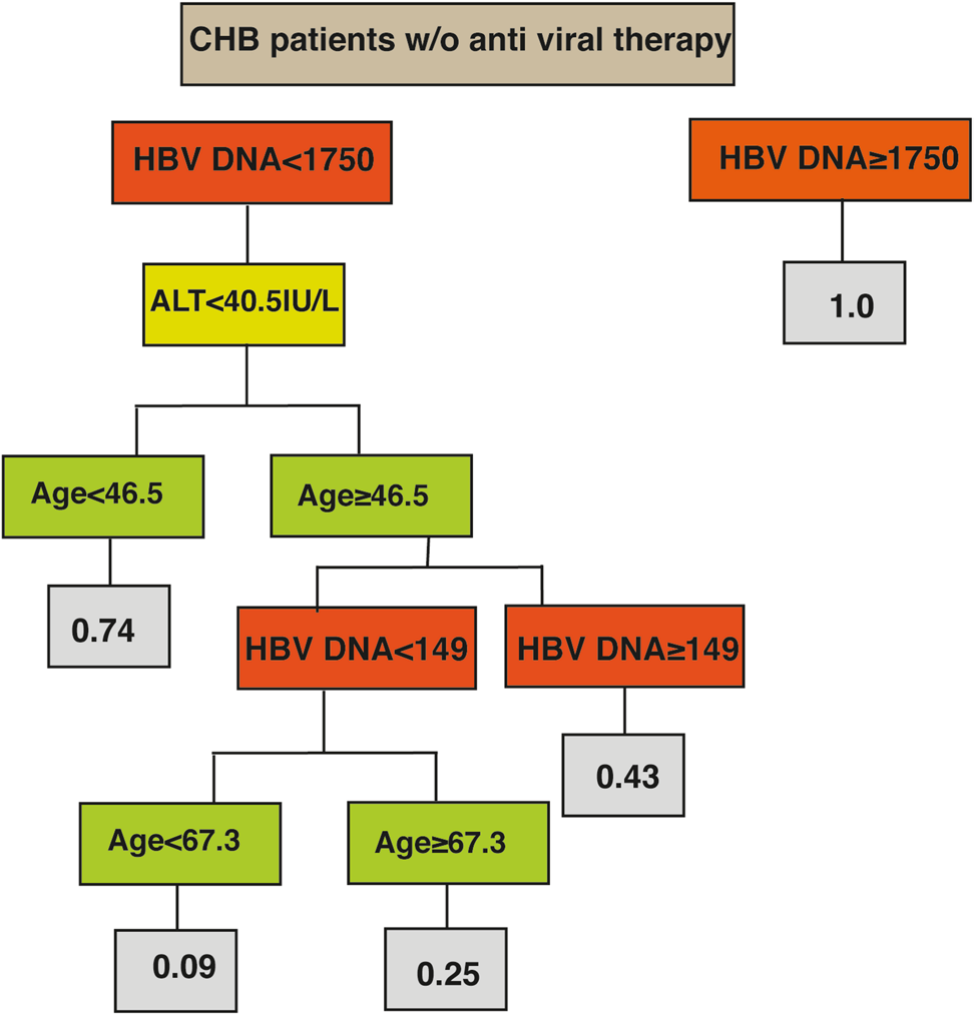
A forest decision tree model, showing the probability of a patient to have an unstable disease course according to individual characteristics. All laboratory results were measured by standard international units (IU/mL).

According to the decision tree model, the most valuable baseline parameters predicting the probability of an unstable disease course were baseline VL, ALT and patients’ age (Fig. 2). Accordingly, a patient between the ages of 46 and 67 years old, with a baseline VL < 149 IU/mL and ALT < 40 IU/L, have a probability of only 9% to have an unstable course of CHB during an average follow-up of 4.4 years. Contrary to this stable pathway, a patient younger than 46 will have a 74% risk of having an unstable disease, even with a baseline ALT of < 40 and VL < 1750 IU/ML.

**Figure 3:**
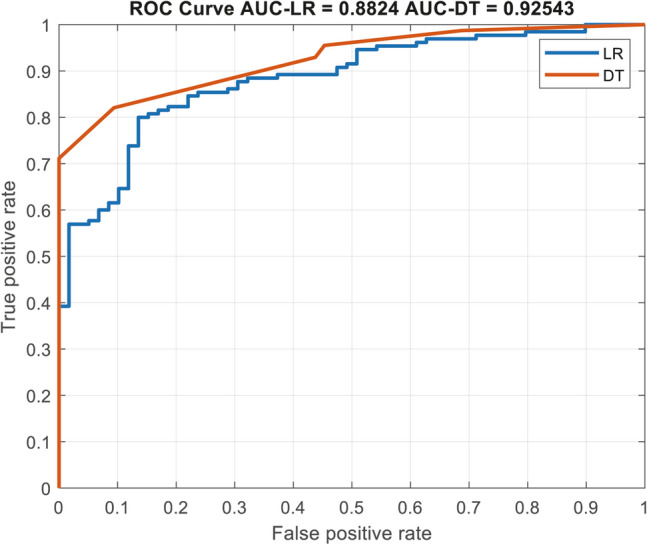
A ROC curve, comparing the validation of both, the multivariate logistic regression (marked as LR) and the decision tree model (marked as DT).

We next calculated the performance of both, the stepwise logistic regression and the decision tree models for the prediction of an unstable disease course among patients in our cohort. As shown in Fig. 3, the logistic regression and the decision tree model had an AUC of 0.88 and 0.92, respectively, in predicting disease stability over an average of 4.4 years of follow-up.

For internal validation, we tested our model by creating a train and test groups. We randomly divided the whole cohort (220 patients) into two groups: a train group which contained 80% of the cohort (176 patients), on which we executed the multivariate logistic regression and the decision tree model; a test group which contained the rest of the cohort (44 patients), on which the results were tested.

**Figure 4:**
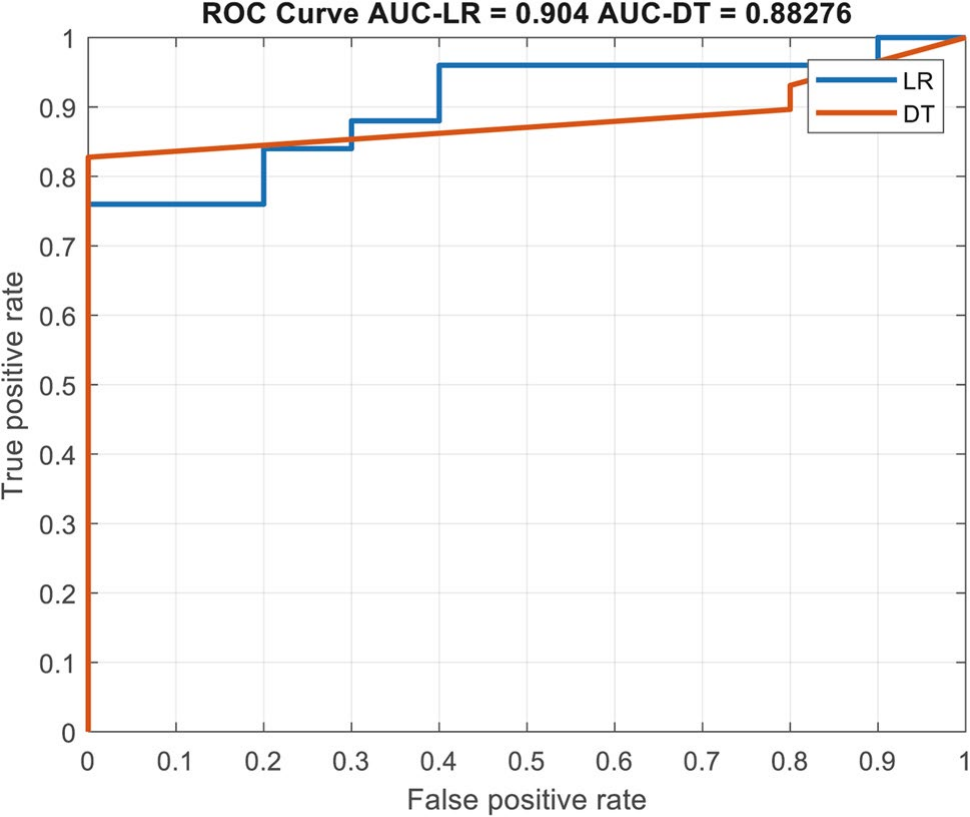
A ROC curve of the test sample, comparing the validation of both, the multivariate logistic regression (marked as LR) and the decision tree model (marked as DT).

As shown in Fig. 4, the test group sample of the logistic regression and the decision tree model had an AUC of 0.9 and 0.88, respectively, in predicting disease stability. In the tenfold cross validation, the mean AUC of the test group was 0.89 (range between 0.72 and 0.97) and the mean error (when we used 0.5 as a threshold value) was 20% (range between 14 and 32%).

## Discussion

Most patients with CHB infection who are HBeAg negative, have normal liver enzymes and low VL and are therefore not amenable for anti-viral treatment according to the updated criteria of the international guidelines^10^. Following diagnosis, these patients are monitored for HBV VL and liver enzymes frequently during the first year after diagnosis, in order to rule out significant fluctuations in these parameters, and at least annually thereafter^12^, in order to assess possible disease progression and the necessity of anti-viral therapy. While having a stable disease, these patients are defined as having chronic HBV infection (formally designated inactive carriers), with an excellent long-term prognosis and a low risk for disease progression and HCC, even without anti-viral therapy^13,14^. Furthermore, these patients have a chance for HBsAg loss and functional cure of 0.7–1.9% per year^12,15–17^. However, the minority of patients, mainly these with a higher baseline HBV DNA and ALT levels, might undergo spontaneous (i.e. with no identifiable causative factor, such as immune suppression treatment) viral reactivation and/or develop liver inflammation and fibrosis during the course of their disease. For example, a study from Greece has shown that among inactive CHB patients, there is a 15% chance of progression to chronic hepatitis status over a 3 year of follow-up^18^. Therefore, a strict follow-up with a frequent monitoring of serum HBV DNA and ALT levels should be undertaken, in order to identify patients who undergo a transition from chronic infection to chronic hepatitis state in a timely manner, and to initiate anti-viral treatment in order to avoid disease progression. However, unselected monitoring is costly and time consuming, and therefore better predictors for disease stability are needed.

In this study, we have analyzed a cohort of 220 HBeAg negative CHB patients without anti-viral treatment in order to identify predictors for disease stability over a course of 4.4 ± 2.2 years of follow-up. We have used stringent criteria for disease stability, that were defined as ALT < 40 and VL < 2000IU/ML during all 6 sequential visits to the liver clinic during follow-up time. Therefore, the high percentage of patients with an unstable disease (156/220, 71%) is not surprising, and most importantly, only 77/156 (49%) of unstable patients were ultimately put on anti-viral therapy.

We have found that baseline ALT, VL and age are the most important predictive parameters for a stable disease. In multivariate analyses, using both logistic regression and decision tree models, we show that incorporating these three parameters can predict, in a more precise manner, disease stability and can identify, in a high accuracy, patients at risk for an unstable disease course.

Short intervals between follow up clinic visits and frequent blood tests are costly and time consuming and even unavailable in many low income countries^19^. We argue that some of these expenditures are unnecessary or at least redundant when tests are taken so frequently in cases of subpopulation whose CHB has a high chance to remain stable over time.

According to our results, older age was found as a predictor for a stable disease. Although aging is associated with more co-morbidities, drug utilization and waning of the immune system^20,21^, we speculate that older patients whose disease was underdiagnosed for many years and have a lower baseline liver enzymes and VL, are more likely to remain stable over the following years. Another element that should be considered is the risk of HCC, which increases with older age^22,23^. The increase prevalence of HCC with older age is in correlation with our decision tree which shows a minimal risk for an unstable disease between ages 46–67 but afterwards, a substantial risk increment for disease instability. The higher risk of HCC and loss of VL stability with age progression may not be linked with a common pathogenesis but justifies a more cautious approach regarding the elderly population with CHB that requires more frequent follow ups.

Male gender was found as a predictor for an unstable disease in the univariate regression analysis. Although there was a mild overrepresentation of males in our research (57% vs 43% M/F), we argue that this small deviation cannot explain the substantial ratio and statistical significance. Therefore, one could speculate that gender diversity in HBV gene expression and replication, previously shown in several studies involving animal models^24^, may be mechanistically linked to our observation. Therefore, patients’ gender may be taken into consideration in the evaluation of CHB patients’ chance to have a stable disease course over time.

Interestingly, the percentage of patients with liver steatosis during follow-up (per ultrasonography) did not differ between the stable vs. unstable groups, although previous reports support a negative correlation between liver steatosis and HBV viral load^25^. We speculate that although liver steatosis may be associated with a lower VL, it also might be associated with higher ALT level, therefore neutralizing any potential effect on disease stability according to our definitions.

Our study has some important limitations that should be considered; First, it is based on a cohort of patients treated at the largest liver clinic located in a tertiary transplantation center in the country, and therefore is amenable to selection bias towards patients with a higher degree of disease severity.

In addition, the study population included only HBeAg negative patients, for whom ALT level, as well as VL might fluctuate frequently over time and affect their eligibility for anti-viral treatment. Further studies should analyze the follow-up frequency required in HBeAg positive patients, who are not eligible to anti-viral treatment under usual circumstances.

Another concern was information bias. However, the data collected in our study was mainly basic demographic and laboratory values derived from electronic records, that are generally not amenable to major mistakes.

Another limitation was that our final analysis is based on a cohort of only 220 patients out of the 579 screened, due to our rigorous study inclusion criteria. However, the sample size was large enough for our analyses, although larger studies are still needed to further verify our conclusions.

Another limitation was that some parameters relevant to a physicians’ decision regarding patients’ follow-up intervals, were not taken into account. Parameters such as patients’ compliance, accessibility to a medical institution, socioeconomic status and patients’ insight of his or her disease were not detectable or were hard to gather. These limitations were not dealt with within the scope of our study. In addition, some laboratory parameters such as quantitative HBsAg and HBcrAg were not routinely tested in our cohort and therefore were not included in the study, despite their potential correlation with disease stability. Further studies should clarify these potential correlations.

In summary, we have created a simple model by which the treating hepatologist can characterize and predict the chance of a CHB patient to have a stable disease course, therefore necessitating less frequent follow up visits. We speculate that this group of patients can be monitored less frequently safely, thereby saving money and withholding unnecessary tests from patients. We acknowledge that certain populations are at higher risk for disease progression and should not go through a stability allocation, such as cirrhotic patients, non-compliant patients or patients on immunosuppressive therapy. Further prospective studies on a larger patient population should be done to validate our findings.

## Data Availability

Anonymous data of this work will be available upon request to Prof. Amir Shlomai at: shlomaiamir@gmail.com.
